# Case, Showing with What Caution Speculative Chemical Testimony Ought to Be Received

**Published:** 1846-12

**Authors:** 


11. *Case, showing with what Caution Speculative Chemical Testimony ought to be received.*

TO THE EDITOR OF THE LANCET.

Sir,—Knowing the readiness with which you always give publicity to facts, the propagation of which may be of essential service, and especially those connected with the medical prefession, I take the liberty of enclosing to you for publication, under the above heading, the following particulars, which came under my observation at the trial of Mary North, at the Surrey Assizes, at Guildford, on the 1st Nov. on a charge of murdering an infant, named Mary Ann Barker, on which occasion I acted as attorney for the prisoner. The chemist alluded to was Mr. Alfred Swaine Taylor, lecturer on medical jurisprudence at Guy's Hospital; and the surgeon was Mr. Tatham, of Wandsworth. From experiments which I had previously made, in order to convince my own mind, I became aware of the difference in colour which would arise upon mixing the ingredients in a different order; and I instructed Mr. Locke, my counsel, to insist upon the experiment being repeated in court in the manner it was. The girl was acquitted, I do not hesitate to say, in consequence of it.

I have the honor to be, Sir,
Your obedient Servant,
JOHN J. HARRINGTON.

At the recent trial of a young woman, on a charge of the murder of an infant, by administering vitriol to it, the prisoner's defence rested entirely upon this question,—whether sulphuric acid, sugar, and water, mixed together in certain proportions; and aniseed, sugar, and water, mixed together in like proportions, would in point of color, a few minutes afterwards, produce a similar, or a somewhat similar appearance. The girl's life undoubtedly hung upon the result. It was the only link wanting to establish her complete defence. If the same result as to appearances followed upon the mixing both sets of ingredients, it established the girl's innocence. If a palpably different color and appearance were the result, that must go very far towards establishing the presumption of her guilt.

The question was put to an eminent surgeon, and his answer was, that they would produce similar, or nearly similar, appearances. After he had given his evidence, and whilst the trial was proceeding, a doubt arising in his mind, he left the court with one of the most distinguished chemists of the day, also a witness at the trial; and they together made the experiment. They returned into court, and the surgeon, in a state of excitement, requested the judge to allow him to correct his testimony; for he found, upon trial, that the two mixtures produced very different appearances: and he held up, in court, two wine glasses, one with the aniseed mixture, which was of a yellow color—the other with the sulphuric acid mixture, which was intensely black.

He was then asked by the counsel for the prisoner in what order he mixed the ingredients producing the black appearance; and he replied, the sulphuric acid first, then the sugar, and then the water; and he was then requested to repeat the experiment in court, putting first the sulphuric acid, then the water, and then the sugar. What was the consequence? Why, instead of turning black, it remained of a yellow color, and as nearly as possible resembled the mixture of aniseed, &c.

Had the experiment not been repeated in court, in the order of mixing the ingredients in which it was there repeated, the girl, in all probability, would have been hanged!

The quantity experimented with was about a tea-spoonful of sulphuric acid to two of water, and a small piece of sugar.

*** Mr. Harrington is entitled to great credit for the tact and ability which he displayed in this case. The life of the innocent accused person was saved by the sagacity of the attorney, and not by the eloquence of the counsel.—Ed. Lancet.

---

*Subnitrate of Bismuth in Gastralgia.*—Dr. Bertini, of Turin, has lately employed this medicine in numerous cases of gastralgia, beginning with a dose of five centigrammes (= 0.77 grains), and gradually raising it to twenty centigrammes (= 3.08 grains). It was mixed with calcined magnesia, and given three or four times a day. In most instances the neuralgic pains disappeared under the use of this medicine: and in cases where the gastralgia appeared to be connected with organic disease, the symptoms were always much alleviated.

